# Textural features reflecting local activity of the hippocampus improve the diagnosis of Alzheimer’s disease and amnestic mild cognitive impairment: A radiomics study based on functional magnetic resonance imaging

**DOI:** 10.3389/fnins.2022.970245

**Published:** 2022-08-08

**Authors:** Luoyu Wang, Qi Feng, Xiuhong Ge, Fenyang Chen, Bo Yu, Bing Chen, Zhengluan Liao, Biying Lin, Yating Lv, Zhongxiang Ding

**Affiliations:** ^1^Key Laboratory of Clinical Cancer Pharmacology and Toxicology Research of Zhejiang Province, Department of Radiology, Affiliated Hangzhou First People’s Hospital, Zhejiang University School of Medicine, Hangzhou, China; ^2^The Center for Integrated Oncology and Precision Medicine, Affiliated Hangzhou First People’s Hospital, Zhejiang University School of Medicine, Hangzhou, China; ^3^The Fourth School of Medical, Zhejiang Chinese Medical University, Hangzhou, China; ^4^School of Medical Imaging, Hangzhou Medical College, Hangzhou, China; ^5^Jing Hengyi School of Education, Hangzhou Normal University, Hangzhou, China; ^6^Center for Rehabilitation Medicine, Department of Geriatric VIP No. 3, Department of Clinical Psychology, Zhejiang Provincial People’s Hospital, Hangzhou, China; ^7^Center for Cognition and Brain Disorders, The Affiliated Hospital of Hangzhou Normal University, Hangzhou, China

**Keywords:** Alzheimer’s disease, amnestic mild cognitive impairment, resting-state functional magnetic resonance imaging, the amplitude of low frequency fluctuation, radiomics

## Abstract

**Background:**

Textural features of the hippocampus in structural magnetic resonance imaging (sMRI) images can serve as potential diagnostic biomarkers for Alzheimer’s disease (AD), while exhibiting a relatively poor discriminant performance in detecting early AD, such as amnestic mild cognitive impairment (aMCI). In contrast to sMRI, functional magnetic resonance imaging (fMRI) can identify brain functional abnormalities in the early stages of cerebral disorders. However, whether the textural features reflecting local functional activity in the hippocampus can improve the diagnostic performance for AD and aMCI remains unclear. In this study, we combined the textural features of the amplitude of low frequency fluctuation (ALFF) in the slow-5 frequency band and structural images in the hippocampus to investigate their diagnostic performance for AD and aMCI using multimodal radiomics technique.

**Methods:**

Totally, 84 AD, 50 aMCI, and 44 normal controls (NCs) were included in the current study. After feature extraction and feature selection, the radiomics models incorporating sMRI images, ALFF values and their combinations in the bilateral hippocampus were established for the diagnosis of AD and aMCI. The effectiveness of these models was evaluated by receiver operating characteristic (ROC) analysis. The radiomics models were further validated using the external data from the Alzheimer’s Disease Neuroimaging Initiative (ADNI) database.

**Results:**

The results of ROC analysis showed that the radiomics models based on structural images in the hippocampus had a better diagnostic performance for AD compared with the models using ALFF, while the ALFF-based model exhibited better discriminant performance for aMCI than the models with structural images. The radiomics models based on the combinations of structural images and ALFF were found to exhibit the highest accuracy for distinguishing AD from NCs and aMCI from NCs.

**Conclusion:**

In this study, we found that the textural features reflecting local functional activity could improve the diagnostic performance of traditional structural models for both AD and aMCI. These findings may deepen our understanding of the pathogenesis of AD, contributing to the early diagnosis of AD.

## Introduction

Alzheimer’s disease (AD) is an aging-related central nervous system disease characterized by impaired memory function, which severely affects the quality of life of the elderly (Masters et al., 2015; Soria Lopez et al., 2019). Recent projection data suggests that, by 2050, the prevalence of dementia will double in Europe and triple globally, and the estimated number of new dementia cases would be three times higher based on the biological rather than clinical definition of Alzheimer’s disease (Knopman et al., 2021; Scheltens et al., 2021). AD is still incurable due to incomplete understanding of its etiology and underlying neurological mechanisms (Sun et al., 2018). However, recent studies have indicated that certain necessary interventions such as statins in the early stages of the disease may slow the progression of AD, prolonging the lifespan of patients (Sperling et al., 2011; McDade and Bateman, 2017). Amnestic Mild Cognitive Impairment (aMCI), characterized by some degree of cognitive decline and memory impairment, is generally considered an early AD (Bradfield et al., 2018). Dietary intervention and alleviation of neuropsychiatric symptoms may reduce the risk of conversion to dementia (Cooper et al., 2015). However, the medical diagnosis of aMCI, which mainly relies on neuropsychological tests, remains challenging due to the lack of objective biological approaches (Murayama et al., 2013; Alves et al., 2021). Therefore, our current research focused on the identification of brain-imaging surrogate markers sensitive to early disease that could distinguish AD from normal cognition in the elderly, thus enabling an efficient effective diagnosis of aMCI.

The hippocampus plays a crucial role in human cognition, especially memory, and it is considered to be the most vulnerable region during AD pathogenesis (Braak and Braak, 1997). Both amyloid-β and Tau proteins have been noted to be selectively deposited in the hippocampal cortical layers of AD patients (Braak and Braak, 1997). In addition, hippocampal gray matter atrophy is an important indicator for assessing the severity of dementia (Pini et al., 2016). Using structural MRI, a previous study showed that a reduction in bilateral hippocampal gray matter volume was associated with cognitive decline in AD and aMCI patients (Feng F. et al., 2021). However, the volumetric measures may overlook some specific morphological features, such as the textural features of the hippocampus (Dachena et al., 2019; Curado et al., 2020).

Radiomics, originally developed for tumor diagnosis, is a computer-aided diagnostic approach used to mine and analyze quantitative image characteristics such as intensity and textural features (Feng Q. et al., 2021; Iancu et al., 2021). Radiomics have been well-validated in the classification of AD and NC based on textural features of the structural hippocampus in previous studies (Rajeesh et al., 2017; Zhao et al., 2020). For example, a previous study has indicated that the accuracy of discrimination of Alzheimer’s disease patients is 93.6% using textural features of the structural hippocampus (Rajeesh et al., 2017). In addition, the hippocampal texture was superior to volume reduction as a predictor of MCI-to-AD conversion (Sørensen et al., 2016; Zhao et al., 2020), though it has been reported that textural features of the structural hippocampus are unsatisfactory in diagnosing aMCI (Feng et al., 2019; Park et al., 2021). This may be because the structural images could not capture all the changes in the hippocampus in aMCI (Cai et al., 2017).

As an advanced non-invasive neuroimaging technique, resting-state functional magnetic resonance imaging (fMRI) is an important imaging modality to understand the neurodegenerative course of aMCI and early AD (Wu et al., 2022), because the memory dysfunction may occur before the structural degeneration (Jin et al., 2012). The amplitude of low frequency fluctuation (ALFF) is proposed to characterize the local properties of rs-fMRI signals (Zang et al., 2007), showing frequency-dependent pattern (Zuo et al., 2010) and temporal variability (dynamics) (Liao et al., 2019), and thus has been widely used to detect functional abnormalities in brain disorders (Li et al., 2020; Wang et al., 2021). As for AD and aMCI patients, previous studies have consistently observed the alterations of ALFF value in the hippocampus (Liu et al., 2014; Cha et al., 2015; Yang et al., 2018; Yuan et al., 2021). For example, Liu and colleagues have demonstrated increased ALFF values in the bilateral hippocampus of AD patients compared with healthy controls (Liu et al., 2014). Meta-analyses have also shown significant alterations of ALFF in the left hippocampus/parahippocampal gyrus in AD and aMCI patients (Cha et al., 2015; Yuan et al., 2021). More importantly, these ALFF changes were found to exhibit a frequency-dependent pattern (Han et al., 2011; Liu et al., 2014). In our previous study, we observed the difference in ALFF in the slow-5 frequency band between groups, mainly corresponding to the bilateral hippocampus as well as regions within the default mode network, with the highest accuracy in discriminating the three groups (Wang et al., 2021). Our findings indicated that ALFF in the slow-5 frequency band might serve as a promising functional indicator to aid the diagnosis of AD and aMCI (Wang et al., 2021). Recently, ALFF combined with structural features has been investigated for the diagnosis of AD and aMCI (Khatri and Kwon, 2022; Liu et al., 2022). Yet ALFF has not been used in the analysis of radiomics and it is unclear whether incorporating functional measures into radiomics analysis can improve the effectiveness of traditional hippocampal structural models for the diagnosis of AD and aMCI.

In this study, we combined the ALFF textural feature of the hippocampus in the slow-5 frequency band with structural MRI images to investigate their discriminative performance for AD and aMCI using radiomics analysis. We hypothesized that the inclusion of hippocampal functional metrics in radiomics could improve the effectiveness of traditional hippocampal structural models in distinguishing AD and aMCI patients from healthy elderly, especially for the diagnosis of aMCI.

## Materials and methods

### Participants

**FIGURE 1:**
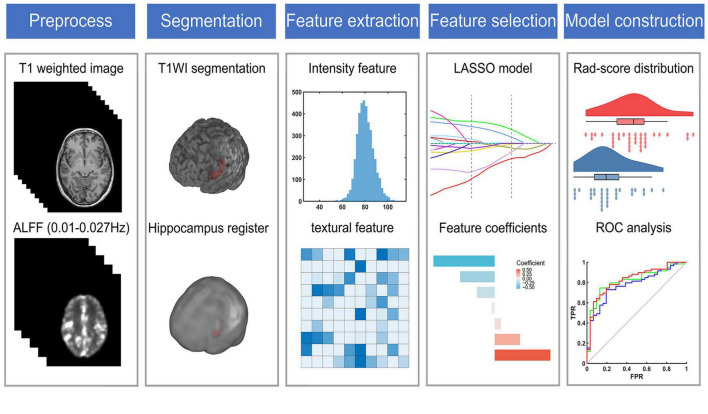
The flow chart of radiomic analysis. ALFF, the amplitude of low frequency fluctuation; LASSO, the least absolute shrinkage and selection operator; ROC, receiver operating characteristic.

From September 2016 to August 2020, 98 AD and 53 aMCI patients at Zhejiang Provincial Hospital and 50 normal controls (NCs) at the hospital’s health promotion center were recruited. All participants signed the written informed consent. This study was approved by the local Ethics Committee of Zhejiang Provincial People’s Hospital (No. 2012KY002) and was conducted according to the Declaration of Helsinki. The inclusion and exclusion criteria have been described at our previous study (Wang et al., 2021). All participants underwent medical history collection, physical examinations, laboratory examinations, routine brain magnetic resonance scans and the Mini-Mental State Test (MMSE). AD patients were diagnosed based on the criteria of the revised NINCDS-ADRDA (National Institute of Neurological and Communicative Disorders and Stroke and the Alzheimer’s Disease and Related Disorders Association) and the DSM-IV-R (revised Diagnostic and Statistical Manual of Mental Disorders, Fourth Edition) with MMSE score ≤ 24. The aMCI patients were selected according to the following criteria: (1) complaint of memory impairment; (2) normal clinical manifestations; (3) 24 < MMSE score ≤ 27; and (4) failure to meet the criteria for dementia according to DSM-IV-R. The inclusion criteria for NCs was as follows: (1) absence of neurological impairment, such as visual loss or hearing and (2) MMSE score ≥ 28. Patients and participants with stroke, brain trauma, epilepsy, Parkinson’s disease, hypertension, serious anemia, diabetes, brain tumor, history of mental illness and signal alterations in the medial temporal cortex caused by infectious or vascular factors on MRI FLAIR and T2-weighted images were excluded. The summary of subjects was illustrated in Table 1 and the flow chart of the radiomic analysis was shown in Figure 1.

**TABLE 1:** Demographic data and clinical characteristics of the participants.

Sample size	AD (*N* = 84)	aMCI (*N* = 50)	NC (*N* = 44)	Statistic	*P*-value
Gender (male: female)	37:47	27:23	21:23	1.244	0.537^a^
Age (years, mean ± SD)	69.226 ± 9.303	65.840 ± 11.171	65.477 ± 9.690	2.847	0.061^b^
Education (years, mean ± SD)	7.167 ± 4.412	7.120 ± 4.059	7.114 ± 3.356	0.003	0.997^b^
MMSE	17.512 ± 5.084	26.200 ± 0.881	29.023 ± 0.902	182.686	<0.001^b^

^a^p-values for sex distribution obtained by the chi-square test; ^b^p-value obtained by analysis of variance. AD, Alzheimer’s disease; aMCI, amnestic mild cognitive impairment; NCs, normal controls.

### Image acquisition

MRI data were obtained using a 3.0T magnetic resonance scanner (Discovery MR750; GE Healthcare, Waukesha, WI, United States) at Zhejiang People’s Hospital and an 8-channel phased array coil was used for all the subjects. Raw structural images were acquired using a high-resolution 3D T1-weighted magnetization-prepared rapid gradient echo (MPRAGE) sagittal sequence with predefined direct MR acquisition parameters [repetition time (TR) = 6.7 ms, echo time (TE) = 2.9 ms, slice thickness = 1 mm, field of view (FOV) = 256 × 256 mm^2^, flip angle = 12^°^, resolution = 256 × 256, and 192 slices]. Rs-fMRI images were acquired using an echo-planar imaging sequence (TR = 2,000 ms, TE = 30 ms, slice thickness = 3.2 mm, FOV = 220 × 220 mm^2^, flip angle = 90^°^, resolution = 64 × 64, and 210 volumes and 44 slices).

### Amplitude of low frequency fluctuation calculation

Rs-fMRI data were mainly processed using SPM^1^ (Penny et al., 2011) and DPABI (Yan et al., 2016) in the following steps: (1) Due to the magnetic field inhomogeneity of the MR machine during the initial scan, the first 10 time points which recommended in DPABI (Chao-Gan and Yu-Feng, 2010) were discarded to reduce the impact on data quality. (2) A temporal layer correction was performed to rectify the differences in interlayer acquisition time using the middle slice as the reference slice. The correction was performed by lagging (shifting forward) the time series on each slice using sinc interpolation. (3) A head motion correction was performed to reduce the effect of the subject’s head motion on data quality (Friston et al., 1995; Whitfield-Gabrieli and Nieto-Castanon, 2012). (4) Linear trend of the time series was regressed. (5) Regression of covariates including white matter, cerebrospinal fluid and Friston 24 parameters was performed (Friston et al., 1996). (6) According to our previous studies (Wang et al., 2021), the ALFF in the slow-5 frequency band (0.01–0.027 Hz) was calculated for each participant. The flow chart for calculating the ALFF in the slow-5 frequency band was shown in Supplementary Figure 1.

In this study, we first excluded patients with the criteria of displacement > 3 mm and rotation > 3°. To improve the reliability of fMRI-based radiomics, we further removed the patients with FD > 0.5 (Power et al., 2015). A 25 subjects were excluded, leaving 84 AD patients, 50 aMCI patients and 44 NCs healthy controls in the follow-up analysis.

### Hippocampus segment

To improve the segmentation efficiency while ensuring the stability of the results, a deep learning-based hippocampal segmentation toolkit hippodeep^2^ was used to automatically segment the bilateral hippocampus (Thyreau et al., 2018). Structural MRI was performed on all patients to obtain bilateral structural image masks of the hippocampus. We randomly selected five cases to compare the segmentation mask of the algorithm and that of a highly qualified head and neck radiologist using the dice coefficient. The mean dice coefficient of the left hippocampus is 0.935 and that of the right hippocampus is 0.967. The results showed the good consistency and validity of the automatic segmentation adopted in our study. Then the bilateral hippocampal masks for assessment of ALFF were obtained by aligning the structural images with the functional images.

### Features extraction

Based on the segmentation results, radiomics features of bilateral hippocampus extracted from two modalities (sMRI and ALFF images) were compared to quantify tissue spatial heterogeneity. The features were analyzed using an open-source radiomics analysis package^3^ based on the radiomics toolbox^4^, conforming to the Imaging Biomarker Standardization Initiative (IBSI) (Xu et al., 2020; Zwanenburg et al., 2020). In the current study, 101 features were extracted from sMRI or ALFF images within each region of the bilateral hippocampus, including 13 intensity features and 88 textural features for each modality (Xu et al., 2020). The names of the 101 radiomic features were shown in Supplementary Table 1.

### Feature selection and radiomic signature building

Before data processing, the createDataPartition function from the caret package was used to randomly split the data of 84 AD patients and 44 NCs, of which 70% of the data were classified as the training set and 30% of the data were classified as the test set and make the ratio of positive samples to negative samples the same between the training and test set. To avoid sample bias of grouping and get a steady result, 10 times repetition of the validation in the present study was adopted. To remove the unit limit of each feature before applying it to the machine learning model for classification, z-normalization was performed on the training set and applied to the test set and the external validation set. Due to sample imbalance, the synthetic minority over-sampling (SMOTE) algorithm (Chawla et al., 2002) was used to balance the minority group in the training set.

Two feature selection methods, including the minimum redundancy maximum relevance (mRMR) (Ding and Peng, 2005) and the least absolute shrinkage and selection operator (LASSO) (Tibshirani, 2011), were used to select the most valuable predictive features in the training cohort. Firstly, using the mRMR method, the features were ranked by their relevance-redundancy index, and the top 20 features with the highest relevance were selected (Ding and Peng, 2005). Then, LASSO regression was conducted on the training cohort using 10-fold cross-validation to choose the optimized subset of features and build a radiomic signature (Tibshirani, 2011). The corresponding coeficients were evaluated. As a simple score developed to classify the patients and NCs using radiomics, the radiomics score (radscore) was calculated by summing selected textural features weighted by their respective coeficients (plus a constant term) (Zheng et al., 2018). All rad-scores between the AD and NCs groups were compared on the training and validation sets, respectively.

The above process was carried out six times in total. Using the same train-test split, six radiomics signatures were created based on sMRI and ALFF in the slow-5 band and their combination in the left and right hippocampus, respectively. Then images from 50 aMCI patients and 44 NCs were similarly processed. The following 12 radiomics signatures were constructed: AD diagnosis model based on left hippocampal structural image, AD diagnosis model based on left hippocampal ALFF, AD diagnosis model based on left hippocampal structural and ALFF image, AD diagnosis model based on right hippocampal structural image, AD diagnosis model based on right hippocampal ALFF, AD diagnosis model based on right hippocampal structural and ALFF images, aMCI diagnosis model based on left hippocampal structural image, aMCI diagnosis model based on left hippocampal ALFF, aMCI diagnosis model based on left hippocampal structural and ALFF images, aMCI diagnosis model based on right hippocampal structural image, aMCI diagnosis model based on right hippocampal ALFF, and aMCI diagnosis model based on right hippocampal structural and ALFF images.

### Statistical analysis

Wilcoxon test was performed on the rad-score for detecting AD and aMCI in the train and test sets, respectively. As recommended in previous study (Ge et al., 2022), *P* < 0.05 was considered to be statistically significant in accordance with statistical conventions. The area under the curve (AUC) of the training and test set was used to assess the discriminative accuracy of the Rad-score. This process was repeated 10 times and the average AUC value was obtained as the final metric for this study. Receiver operating characteristic (ROC) curves were analyzed and visualized using the Matlab-based classification model effectiveness analysis tool ROCA.^5^ To further assess the classification effects of different models, this study used the Delong test (DeLong et al., 1988) to compare the differences in the AUCs of each classification model.

Correlation analyses were performed on the features retained for AD and aMCI diagnosis. The textural features selected in unimodality and retained in the combined model were correlated with MMSE by the Spearman correlation coefficient, and an α level of less than 0.05 was considered statistically significant. The correlation coefficient was calculated using the following formula:


$$\rho =\frac{\sum_{i}\left(R\,\left(x_{i}\right)-R\,\left(\bar{x}\right)\right)\,\left(R\,\left(y_{i}\right)-R\,\left(\bar{y}\right)\right)}{\sqrt{\sum_{i\,{\left(R\,\left(x_{i}\right)-R\,\left(\bar{x}\right)\right)}^{2}}}\,\sqrt{\sum_{i\,{\left(R\,\left(y_{i}\right)-R\,\left(\bar{y}\right)\right)}^{2}}}}$$

Where, R(x) and R(y) are the rank order of x and y, respectively.

### External validation

The external validation dataset including 33 AD, 34 MCI patients and 38 NCs was downloaded from the ADNI database.^6^ The searching criteria were as follows: (1) data containing 3.0 T Philips MRI scans; (2) scan sequences containing high-definition T1 structural images; (3) scan sequences containing resting-state functional MRI data (TR = 3.0 s, layer thickness = 3.3 mm, resolution = 64 × 64, and 140 time points); and (4) the baseline data were collected from the initial visit. ADNI was reviewed and approved by the institutional review boards of all participating institutions^7^, and written informed consent was obtained from all participants or their guardians in accordance with the Declaration of Helsinki (Petersen et al., 2010; Trojanowski et al., 2010; Weiner et al., 2010). A total of 6 subjects with a maximum head movement displacement > 3 mm, a rotation > 3° and an FD > 0.5 during resting-state functional MRI scanning were excluded, and 32 AD, 32 MCI, and 35 NCs subjects were finally included in the validation analyses. The summary of ADNI subjects were shown in Supplementary Table 2.

The radiomics models obtained from the train set of our data were applied to the ADNI dataset to validate the robustness of the models in clinical practice. In addition, to further validate our results, we performed a classification analysis with a combination of the bilateral hippocampus.

## Results

### Demographic data and neuropsychological tests

No significant differences in demographic information (i.e., sex, age, education) were noted (*P* > 0.05). A significant difference in MMSE score was shown among the three groups. *Post hoc* analyses were performed and the results indicated that the NCs had the highest neuropsychological performance, aMCI patients had intermediate performance, AD patients had the worst performance (*P* < 0.001). Table 1 summarizes the detailed demographic characteristics and MMSE scores of all subjects.

### Receiver operating characteristic analysis and delong tests

**FIGURE 2:**
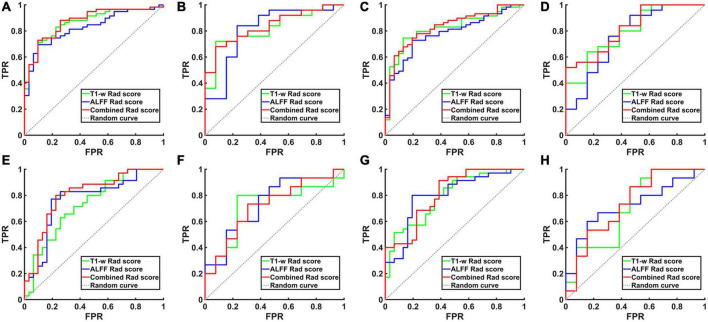
The ROC curve of the hippocampal structural image, hippocampal ALFF in slow-5 frequency band and their combined model. **(A)** ROC curves for AD and NCs in the training set based on left hippocampal images. **(B)** ROC curves for aMCI and NCs in the training set based on left hippocampal images. **(C)** ROC curves for AD and NCs in the training set based on right hippocampal images. **(D)** ROC curves for aMCI and NCs in the training set based on right hippocampal images. **(E)** ROC curves for AD and NCs in the test set based on left hippocampal images. **(F)** ROC curves for aMCI and NCs in the test set based on left hippocampal images. **(G)** ROC curves for AD and NCs in the test set based on right hippocampal images. **(H)** ROC curves for aMCI and NCs in the test set based on right hippocampal images. TPR, true positive rate; FPR, false positive rate; AD, Alzheimer’s disease; aMCI, amnestic mild cognitive impairment; NCs, normal controls; ROC, receiver operating characteristic; ALFF, the amplitude of low frequency fluctuation.

The processes of feature selection and rad-score calculation for all 12 models are shown in Supplementary Figures 2–7. The results of the ROC analyses are shown in Figure 2 and Tables 2, 3.

**TABLE 2:** The ROC curve of left hippocampal structural images, ALFF in slow-5 frequency band and their combined model.

Classifier	Model	Data set	AUC	95% CI	Accuracy
AD vs. NCs	T1	Training	0.864	0.841–0.887	0.778
		Test	0.818	0.767–0.858	0.790
	ALFF	Training	0.828	0.801–0.853	0.767
		Test	0.809	0.758–0.856	0.816
	T1+ALFF	Training	0.873	0.849–0.895	0.789
		Test	0.837	0.792–0.872	0.763
aMCI vs. NCs	T1	Training	0.729	0.689–0.766	0.682
		Test	0.713	0.650–0.772	0.786
	ALFF	Training	0.764	0.725–0.800	0.788
		Test	0.738	0.678–0.796	0.714
	T1+ALFF	Training	0.804	0.763–0.834	0.788
		Test	0.718	0.657–0.774	0.714

AD, Alzheimer’s disease; aMCI, amnestic mild cognitive impairment; NCs, normal controls; ROC, area under the curve; ALFF, the amplitude of low frequency fluctuation.

**TABLE 3:** The ROC curve of right hippocampal structural images, ALFF in slow-5 frequency band and their combined model.

Classifier	Model	Data set	AUC	95% CI	Accuracy
AD vs. NCs	T1	Training	0.818	0.789–0.844	0.788
		Test	0.797	0.750–0.838	0.710
	ALFF	Training	0.780	0.746–0.806	0.756
		Test	0.763	0.708–0.816	0.789
	T1+ALFF	Training	0.830	0.802–0.856	0.778
		Test	0.822	0.772–0.857	0.684
aMCI vs. NCs	T1	Train	0.790	0.756–0.825	0.712
		Test	0.708	0.643–0.772	0.714
	ALFF	Training	0.798	0757–0.830	0.803
		Test	0.723	0.659–0.780	0.714
	T1+ALFF	Training	0.810	0.778–0.840	0.722
		Test	0.733	0.666–0.793	0.714

AD, Alzheimer’s disease; aMCI, amnestic mild cognitive impairment; NCs, normal controls; ROC, area under the curve; ALFF, the amplitude of low frequency fluctuation.

When differentiating the AD from NCs, the AUC of the left hippocampal structural model was 0.864, while that of the model based on left hippocampal ALFF in the slow-5 frequency band was 0.828. Delong test reflected the significant difference in AUC between these two models (*z* = 3.087, *P* = 0.002). The combined model based on the left hippocampal structural and ALFF images exhibited the highest accuracy (AUC = 0.873). And the AUC of the combined model was significantly higher than that of the structural image model (*z* = 3.003, *P* = 0.003). In addition, similar results were obtained for assessing the right hippocampus. The AUC of the right hippocampal structural model was 0.818, while that of the model based on right hippocampal ALFF in the slow-5 frequency band was 0.780. Delong test reflected the significant AUC difference between these two models (*z* = 2.898, *P* = 0.004). The combined model based on the right hippocampal structural and ALFF images exhibited the highest accuracy (AUC = 0.830). Additionally, the AUC of the combined model was significantly higher than that of the structural image model (*z* = 2.361, *P* = 0.018).

Unlike AD, the model based on left hippocampal ALFF in the slow-5 frequency (AUC = 0.764) showed better discriminative performance than the left hippocampal structural model (AUC = 0.729) when distinguishing aMCI from NCs. There was a marginally significant difference in AUC between these two models (*z* = 1.805, *P* < 0.071). The combined model based on the left hippocampal structural and ALFF image (AUC = 0.804) had better performance than the left hippocampal structural model (*z* = 6.629, *P* < 0.001). The combined model based on the right hippocampal structural and ALFF images (AUC = 0.810) also had better performance (*z* = 1.763, *P* = 0.078) than the right hippocampal structural model (AUC = 0.790).

The results of external validation suggested a consistent trend between the validation and the train sets, as shown in Table 4. In addition, we performed a classification analysis with a combination of the bilateral hippocampus. The results were consistent with the findings based on the unilateral hippocampus and were shown in the Supplementary Table 3.

**TABLE 4:** External validation.

Classifier	Model	Hippocampus	AUC	95% CI	Accuracy
AD vs. NCs	T1	left	0.829	0.794–0.859	0.791
		right	0.738	0.700–0.773	0.731
	ALFF	left	0.757	0.719–0.790	0.731
		right	0.678	0.638–0.717	0.657
	T1+ALFF	left	0.830	0.795–0.862	0.791
		right	0.746	0.706–0.781	0.701
MCI vs. NCs	T1	left	0.554	0.511–0.600	0.627
		right	0.529	0.487–0.574	0.582
	ALFF	left	0.598	0.552–0.639	0.642
		right	0.563	0.520–0.611	0.597
	T1+ALFF	left	0.634	0.589–0.676	0.642
		right	0.558	0.512–0.603	0.582

AD, Alzheimer’s disease; aMCI, amnestic mild cognitive impairment; NCs, normal controls; ROC, area under the curve; ALFF, the amplitude of low frequency fluctuation.

### Correlation analysis

**FIGURE 3:**
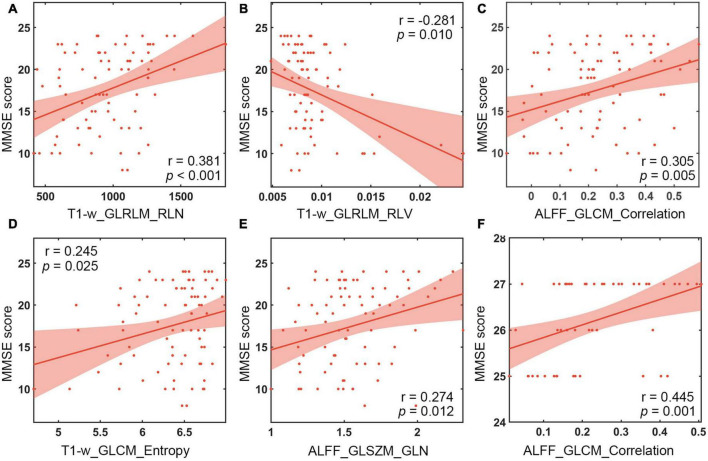
Correlation analysis between MMSE scales and textural features. **(A)** Correlation of T1-w_GLRLM_RLN in the left hippocampus with MMSE scores of AD patients. **(B)** Correlation of T1-w_GLRLM_RLV in the left hippocampus with MMSE scores of AD patients. **(C)** Correlation of ALFF_GLCM_Correlation in the left hippocampus with MMSE scores of AD patients. **(D)** Correlation between T1-w_GLCM_Entropy in the right hippocampus and MMSE scores of AD patients. **(E)** Correlation of ALFF_GLSZM_GLN in the right hippocampus with MMSE scores of AD patients. **(F)** Correlation of ALFF_GLCM_Correlation in the left hippocampus with MMSE scores of aMCI patients. MMSE, Mini Mental State Test; ALFF, the amplitude of low frequency fluctuation.

The features subjected to unimodal selection and retained in the combined model were correlated with the MMSE scores in AD and aMCI diagnostic models, respectively, and the results were shown in Figure 3. In the diagnostic model for AD, the features significantly associated with MMSE score were T1-w_GLRLM (gray-level run-length matrix) _RLN (run-length non-uniformity) (*r* = 0.381, *P* < 0.001), T1-w_GLRLM_RLV (run-length variance) (*r* = −0.281, *P* = 0.012), ALFF_GLCM (gray level concurrence matrix)_Correlation (*r* = 0.305, *P* = 0.005) from the left hippocampus and T1-w_GLCM_Entropy (*r* = 0.245, *P* < 0.025), and ALFF_GLSZM (gray-level size zone matrix)_GLN (gray-level non-uniformity) from the right hippocampus (*r* = 0.274, *P* = 0.010). In the diagnostic model for aMCI, significant MMSE-correlated features included ALFF_GLCM_Correlation from the left hippocampus (*r* = 0.445, *P* = 0.001).

## Discussion

To the best of our knowledge, this was the first study to explore the functional indicator ALFF calculated from rs-fMRI as textural features. In the present study, the textural features of the hippocampus in both ALFF map in the slow-5 frequency band and structural MRI image were combined in the radiomics model to explore their discriminant performance for detecting AD and aMCI. We found that the radiomics model based on hippocampal structural image had a better performance than that based on ALFF in the slow-5 frequency band when distinguishing AD from NCs. When differentiating the aMCI from NCs, the model based on hippocampal ALFF in the slow-5 frequency band showed better diagnostic ability than that based on hippocampal structural images. More importantly, the combined model exhibited the best performance for the diagnosis of both AD and aMCI, which meant that the multimodal radiomics models based on hippocampal structural images and ALFF in the slow-5 frequency band had the potential to become a new diagnostic tool for AD.

Using the radiomics approach, we found that the model based on hippocampus structural images performed well in diagnosing AD. This was consistent with the results of previous radiomics studies on hippocampal structural MRI (Zhang et al., 2012; Rajeesh et al., 2017). Moreover, the above model showed better performance than the model based on hippocampal ALFF in the slow-5 frequency band. On the contrary, the model based on hippocampal ALFF in the slow-5 frequency band instead had better performance than the radiomics model based on radiomics studies of hippocampal structural MRI in diagnosing aMCI. The structural image may not fully reflect the changes in the hippocampus of aMCI patients, and ALFF is generally considered to represent the local activity of the brain (Zang et al., 2007). In the early stages of AD when structural damage is not yet evident, local brain functional changes may precede structural changes. The model based on hippocampal structural images and ALFF in the slow-5 frequency band showed better performance on AD (*z* = 3.003, *P* = 0.003, for left hippocampus; *z* = 2.361, *P* = 0.018, for right hippocampus) and aMCI (*z* = 6.629, *P* < 0.001, for left hippocampus; *z* = 1.763, *P* = 0.078, for right hippocampus) than the classical model based on structural images. The textural features of ALFF may provide additional spatial textural information about the local activity of the brain. In addition, consistent results were also obtained in the external validation set. These results together suggest that the textural features of hippocampal ALFF could improve the diagnosis of traditional structural hippocampus models for AD and aMCI. In contrast, the diagnostic power for aMCI in the validation set was relatively lower than in our data. There are differences in the inclusion criteria such as MMSE scores and symptoms^8^ between the two datasets. In our data, the MMSE score for aMCI patients is between 24 and 27, while in the ADNI database, the MMSE score for aMCI is between 24 and 30. Moreover, aMCI patients from our dataset have a complaint of memory impairment and normal clinical manifestations, while aMCI patients in ADNI suffer a subjective memory concern, informant, or clinician and absence of significant levels of impairment in other cognitive domains. Thus, we speculate that the lower diagnostic power for aMCI in the validation set may be due to the differences in the inclusion criteria. Future studies to recruit the aMCI patients with the same inclusion criteria as our data could be attained to test the radiomics models in the current study.

The features retained in the combined model were correlated with the MMSE score. Our results were consistent with previous studies which showed significant correlations between the run-length non-uniformity (RLN) based on hippocampal structural images and MMSE score (Zhao et al., 2020). In addition, correlation analysis further revealed that the features that were significantly correlated with MMSE scores were T1-w_GLRLM_RLN from the left hippocampus, T1-w_GLRLM_RLV, ALFF_GLCM_Correlation and T1-w_GLCM_Entropy and ALFF_GLSZM_GLN from the right hippocampus in diagnosing AD. Moreover, the feature ALFF_GLCM_Correlation was significantly correlated with MMSE score in detecting aMCI. The features mentioned above may be associated with the cognitive decline of AD or aMCI patients. Among the textural features, ALFF_GLCM_Correlation preserved by feature selection in both diagnosing AD and aMCI was positively correlated with MMSE score (Figure 3). GLCM is generally defined as the joint probability occurrence of pixel or voxel pairs, and GLCM_Correlation is usually considered to reflect the consistency of the image texture (Haralick et al., 1973). The correlation is high if all matrix element values are consistent and low if the values of matrix elements are not consistent. The results of this study were obtained using the grayscale distribution in all 13 directions, reflecting the joint probability information of the image grayscale in the adjacent 26 voxels. GLCM_Correlation may reflect the local texture consistency in the image space (Haralick et al., 1973), and ALFF is generally considered to represent the local activity of the brain (Zang et al., 2007). Therefore, ALFF_GLCM_Correlation may reflect the local coherence of the brain’s activity. This finding suggests that AD and aMCI patients may show cognitive decline as the local coherence decreases. The underlying mechanism is still currently unclear, probably because Tau protein and amyloid-β are selectively deposited in the hippocampal cortex of patients during early onset of AD (Braak and Braak, 1997), resulting in a coherent alteration in the local activity of brain function, which is consistent with previous Regional Homogeneity (ReHo) findings in AD and aMCI patients (Zang et al., 2004; Cha et al., 2015; Wang et al., 2015).

This study had certain shortcomings and limitations. First and foremost, we only used hippocampal ALFF in the slow-5 frequency band, ignoring the consistency within the hippocampus and the connectivity within the whole brain. Future studies should make full use of the advantages of multiple indicators from fMRI such as ALFF, Regional Homogeneity and degree centrality for a comprehensive analysis. Second, the patients with AD and aMCI suffered from cognitive impairments in multiple domains (Stogmann et al., 2016), which could not be fully evaluated by MMSE. However, we failed to collect other cognitive scales and behavior data. Future studies employing different cognitive and behavioral tests in AD patients can aid the validation of the results. Third, the altered hippocampal function may not be the most significant alteration in AD. In future studies, texture features of other brain regions with functional changes, such as the default network, should be employed to further explore their diagnostic effect. Finally, recent studies which adopted a 3-class classification model and exhibited better discriminative performance usually included thousands of data samples (Elola et al., 2021; Katz et al., 2021). We only have 178 MRI data and failed to build a 3-class classification model in the current study. In future study, more data should be acquired to classify AD and MCI simultaneously by building a 3-class classification model.

## Conclusion

In this study, we used the hippocampal radiomics technique to establish predictive models incorporating structural image, ALFF in slow-5 frequency band and their combinations for diagnosis of AD and aMCI. We found that the radiomics model based on hippocampal structural image had a better diagnostic power for detecting AD compared with the model using hippocampal ALFF in the slow-5 frequency band; while the model based on ALFF in the slow-5 frequency band had a higher diagnostic power for aMCI than that based on the hippocampal structural image. The textural features of hippocampal ALFF can improve the diagnostic accuracy of traditional structural image models for detecting AD and aMCI, which meant that multimodal radiomics models based on the hippocampal structural images and the ALFF in the slow-5 frequency band can better diagnose AD and aMCI compared with the traditional structural image model, having the potential to become a new AD diagnostic tool. In future studies, we would make full use of the advantages of multiple indicators from fMRI such as ALFF and functional connectivity, to further examine their diagnostic effect.

## Data availability statement

The raw data supporting the conclusions of this article will be made available by the authors, without undue reservation.

## Ethics statement

The studies involving human participants were reviewed and approved by the Local Ethics Committee of Zhejiang Provincial People’s Hospital. The patients/participants provided their written informed consent to participate in this study.

## Author contributions

ZD and YL: work concept or design and make important revisions to the manuscript. LW: draft manuscript and data processing. QF: collect data. XG, FC, BY, BC, ZL, and BL: provide the measurement devices and review the manuscript. All authors contributed to the article and approved the final manuscript for publication.

## Footnotes

1
http://fil.ion.ucl.ac.uk/spm

2
https://github.com/bthyreau/hippodeep_pytorch

3
https://github.com/WenbingLv/Subregional-Radiomics

4
https://github.com/mvallieres/radiomics

5
https://github.com/Luoyu-Wang/ROCA

6
https://adni.loni.usc.edu

7
http://www.adni-info.org

8
https://adni.loni.usc.edu/methods/documents/

## References

[B1] Alves L. Cardoso S. Silva D. Mendes T. Marôco J. Nogueira J. (2021). Neuropsychological profile of amyloid-positive versus amyloid-negative amnestic mild cognitive impairment. *J. Neuropsychol.* 15(Suppl. 1), 41–52. 32588984 10.1111/jnp.12218

[B2] Braak H. Braak E. (1997). Frequency of stages of Alzheimer-related lesions in different age categories. *Neurobiol. Aging* 18 351–357. 10.1016/s0197-4580(97)00056-0 9330961

[B3] Bradfield N. I. Ellis K. A. Savage G. Maruff P. Burnham S. Darby D. (2018). Baseline amnestic severity predicts progression from amnestic mild cognitive impairment to Alzheimer disease dementia at 3 years. *Alzheimer Dis. Assoc. Disord.* 32 190–196. 10.1097/WAD.0000000000000252 29561277

[B4] Cai S. Chong T. Peng Y. Shen W. Li J. von Deneen K. M. (2017). Altered functional brain networks in amnestic mild cognitive impairment: A resting-state fMRI study. *Brain Imaging Behav.* 11 619–631. 10.1007/s11682-016-9539-0 26972578

[B5] Cha J. Hwang J. M. Jo H. J. Seo S. W. Na D. L. Lee J. M. (2015). Assessment of functional characteristics of amnestic mild cognitive impairment and Alzheimer’s Disease using various methods of resting-state FMRI analysis. *Biomed. Res. Int.* 2015:907464. 10.1155/2015/907464 26180816 PMC4477185

[B6] Chao-Gan Y. Yu-Feng Z. (2010). DPARSF: A MATLAB TOOLBOX for “Pipeline” data analysis of resting-state fMRI. *Front. Syst. Neurosci.* 4:13. 10.3389/fnsys.2010.00013 20577591 PMC2889691

[B7] Chawla N. V. Bowyer K. W. Hall L. O. Kegelmeyer W. P. (2002). Smote: Synthetic minority over-sampling technique. *J. Artif. Intell. Res.* 16 321–357.

[B8] Cooper C. Sommerlad A. Lyketsos C. G. Livingston G. (2015). Modifiable predictors of dementia in mild cognitive impairment: A systematic review and meta-analysis. *Am. J. Psychiatry* 172 323–334. 10.1176/appi.ajp.2014.14070878 25698435

[B9] Curado M. Escolano F. Lozano M. A. Hancock E. R. (2020). Early detection of Alzheimer’s Disease: Detecting asymmetries with a return random walk link predictor. *Entropy* 22:465. 10.3390/e22040465 33286239 PMC7516949

[B10] Dachena C. Casu S. Fanti A. Lodi M. B. Mazzarella G. (2019). Combined use of mri, fmriand cognitive data for Alzheimer’s disease: Preliminary results. *Appl. Sci.* 9:3156.

[B11] DeLong E. R. DeLong D. M. Clarke-Pearson D. L. (1988). Comparing the areas under two or more correlated receiver operating characteristic curves: A nonparametric approach. *Biometrics* 44 837–845. 3203132

[B12] Ding C. Peng H. (2005). Minimum redundancy feature selection from microarray gene expression data. *J. Bioinform. Comput. Biol.* 3 185–205. 10.1142/s0219720005001004 15852500

[B13] Elola A. Aramendi E. Irusta U. Berve P. O. Wik L. (2021). Multimodal algorithms for the classification of circulation states during out-of-hospital cardiac arrest. *IEEE Trans. Biomed. Eng.* 68 1913–1922. 10.1109/TBME.2020.3030216 33044927

[B14] Feng F. Huang W. Meng Q. Hao W. Yao H. Zhou B. (2021). Altered volume and structural connectivity of the hippocampus in Alzheimer’s Disease and amnestic mild cognitive impairment. *Front. Aging Neurosci.* 13:705030. 10.3389/fnagi.2021.705030 34675796 PMC8524052

[B15] Feng Q. Niu J. Wang L. Pang P. Wang M. Liao Z. (2021). Comprehensive classification models based on amygdala radiomic features for Alzheimer’s disease and mild cognitive impairment. *Brain Imaging Behav.* 15 2377–2386. 10.1007/s11682-020-00434-z 33537928

[B16] Feng Q. Song Q. Wang M. Pang P. Liao Z. Jiang H. (2019). Hippocampus radiomic biomarkers for the diagnosis of amnestic mild cognitive impairment: A machine learning method. *Front. Aging Neurosci.* 11:323. 10.3389/fnagi.2019.00323 31824302 PMC6881244

[B17] Friston K. J. Frith C. D. Frackowiak R. S. Turner R. (1995). Characterizing dynamic brain responses with fMRI: A multivariate approach. *Neuroimage* 2 166–172. 10.1006/nimg.1995.1019 9343599

[B18] Friston K. J. Williams S. Howard R. Frackowiak R. S. Turner R. (1996). Movement-related effects in fMRI time-series. *Magn. Reson. Med.* 35 346–355. 10.1002/mrm.1910350312 8699946

[B19] Ge X. Wang L. Pan L. Ye H. Zhu X. Feng Q. (2022). Risk factors for unilateral trigeminal neuralgia based on machine learning. *Front. Neurol.* 13:862973. 10.3389/fneur.2022.862973 35463121 PMC9024101

[B20] Han Y. Wang J. Zhao Z. Min B. Lu J. Li K. (2011). Frequency-dependent changes in the amplitude of low-frequency fluctuations in amnestic mild cognitive impairment: A resting-state fMRI study. *Neuroimage* 55 287–295. 10.1016/j.neuroimage.2010.11.059 21118724

[B21] Haralick R. M. Shanmugam K. Dinstein I. (1973). Textural features for image classification. *Stud. Media Commun.* 3 610–621.

[B22] Iancu R. I. Zara A. D. Mirestean C. C. Iancu D. (2021). Radiomics in head and neck cancers radiotherapy. Promises and Challenges. *Maedica* 16 482–488. 10.26574/maedica.2020.16.3.482 34925606 PMC8643547

[B23] Jin M. Pelak V. S. Curran T. Nandy R. R. Cordes D. (2012). A preliminary study of functional abnormalities in aMCI subjects during different episodic memory tasks. *Magn. Reson. Imaging* 30 459–470. 10.1016/j.mri.2011.12.014 22387024 PMC3327830

[B24] Katz I. O’Brien B. Clark S. Thompson C. T. Schapiro B. Azzi A. (2021). Assessment of a diagnostic classification system for management of lesions to exclude melanoma. *JAMA Netw. Open* 4:e2134614. 10.1001/jamanetworkopen.2021.34614 34889949 PMC8665368

[B25] Khatri U. Kwon G. R. (2022). Alzheimer’s disease diagnosis and biomarker analysis using resting-state functional MRI functional brain network with multi-measures features and hippocampal subfield and amygdala volume of structural MRI. *Front. Aging Neurosci.* 14:818871. 10.3389/fnagi.2022.818871 35707703 PMC9190953

[B26] Knopman D. S. Amieva H. Petersen R. C. Chételat G. Holtzman D. M. Hyman B. T. (2021). Alzheimer disease. *Nat. Rev. Dis. Primers* 7:33. 10.1038/s41572-021-00269-y 33986301 PMC8574196

[B27] Li Z. Li K. Luo X. Zeng Q. Zhao S. Zhang B. (2020). Distinct brain functional impairment patterns between suspected Non-Alzheimer disease pathophysiology and Alzheimer’s Disease: A study combining static and dynamic functional magnetic resonance imaging. *Front. Aging Neurosci.* 12:550664. 10.3389/fnagi.2020.550664 33328953 PMC7719833

[B28] Liao W. Li J. Ji G. J. Wu G. R. Long Z. Xu Q. (2019). Endless fluctuations: Temporal dynamics of the amplitude of low frequency fluctuations. *IEEE Trans. Med. Imaging* 38 2523–2532. 10.1109/TMI.2019.2904555 30872224

[B29] Liu L. Wang T. Du X. Zhang X. Xue C. Ma Y. (2022). Concurrent structural and functional patterns in patients with amnestic mild cognitive impairment. *Front. Aging Neurosci.* 14:838161. 10.3389/fnagi.2022.838161 35663572 PMC9161636

[B30] Liu X. Wang S. Zhang X. Wang Z. Tian X. He Y. (2014). Abnormal amplitude of low-frequency fluctuations of intrinsic brain activity in Alzheimer’s disease. *J. Alzheimers Dis.* 40 387–397. 10.3233/JAD-131322 24473186

[B31] Masters C. L. Bateman R. Blennow K. Rowe C. C. Sperling R. A. Cummings J. L. (2015). Alzheimer’s disease. *Nat. Rev. Dis. Primers* 1:15056. 10.1038/nrdp.2015.56 27188934

[B32] McDade E. Bateman R. J. (2017). Stop Alzheimer’s before it starts. *Nature* 547 153–155. 10.1038/547153a 28703214

[B33] Murayama N. Tagaya H. Ota K. Fujishiro H. Manabe Y. Sato K. (2013). Neuropsychological detection of the early stage of amnestic mild cognitive impairment without objective memory impairment. *Dement. Geriatr. Cogn. Disord.* 35 98–105. 10.1159/000346286 23392179

[B34] Park Y. W. Choi D. Park M. Ahn S. J. Ahn S. S. Suh S. H. (2021). Predicting amyloid pathology in mild cognitive impairment using radiomics analysis of magnetic resonance imaging. *J. Alzheimers Dis.* 79 483–491. 10.3233/JAD-200734 33337361

[B35] Penny W. D. Friston K. J. Ashburner J. T. Kiebel S. J. Nichols T. E. (2011). *Statistical Parametric Mapping: The Analysis of Functional Brain Images.* Amsterdam: Elsevier.

[B36] Petersen R. C. Aisen P. S. Beckett L. A. Donohue M. C. Gamst A. C. Harvey D. J. (2010). Alzheimer’s Disease Neuroimaging Initiative (ADNI): Clinical characterization. *Neurology* 74 201–209. 10.1212/WNL.0b013e3181cb3e25 20042704 PMC2809036

[B37] Pini L. Pievani M. Bocchetta M. Altomare D. Bosco P. Cavedo E. (2016). Brain atrophy in Alzheimer’s Disease and aging. *Ageing Res. Rev.* 30 25–48. 10.1016/j.arr.2016.01.002 26827786

[B38] Power J. D. Schlaggar B. L. Petersen S. E. (2015). Recent progress and outstanding issues in motion correction in resting state fMRI. *Neuroimage* 105 536–551. 10.1016/j.neuroimage.2014.10.044 25462692 PMC4262543

[B39] Rajeesh J. Moni R. S. Gopalakrishnan T. (2017). Discrimination of Alzheimer’s disease using hippocampus texture features from MRI. *Asian Biomed.* 6 87–94.

[B40] Scheltens P. De Strooper B. Kivipelto M. Holstege H. Chételat G. Teunissen C. E. (2021). Alzheimer’s disease. *Lancet* 397 1577–1590. 10.1016/S0140-6736(20)32205-4 33667416 PMC8354300

[B41] Sørensen L. Igel C. Liv Hansen N. Osler M. Lauritzen M. Rostrup E. (2016). Early detection of Alzheimer’s disease using MRI hippocampal texture. *Hum. Brain Mapp.* 37 1148–1161. 26686837 10.1002/hbm.23091 PMC6867374

[B42] Soria Lopez J. A. González H. M. Léger G. C. (2019). Alzheimer’s disease. *Handb. Clin. Neurol.* 167 231–255. 10.1016/B978-0-12-804766-8.00013-3 31753135

[B43] Sperling R. A. Aisen P. S. Beckett L. A. Bennett D. A. Craft S. Fagan A. M. (2011). Toward defining the preclinical stages of Alzheimer’s disease: Recommendations from the National Institute on Aging-Alzheimer’s Association workgroups on diagnostic guidelines for Alzheimer’s disease. *Alzheimers Dement.* 7 280–292. 10.1016/j.jalz.2011.03.003 21514248 PMC3220946

[B44] Stogmann E. Moser D. Klug S. Gleiss A. Auff E. Dal-Bianco P. (2016). Activities of daily living and depressive symptoms in patients with subjective cognitive decline, mild cognitive impairment, and Alzheimer’s Disease. *J. Alzheimers Dis.* 49 1043–1050. 26577522 10.3233/JAD-150785

[B45] Sun B. L. Li W. W. Zhu C. Jin W. S. Zeng F. Liu Y. H. (2018). Clinical research on Alzheimer’s Disease: Progress and perspectives. *Neurosci. Bull.* 34 1111–1118. 10.1007/s12264-018-0249-z 29956105 PMC6246849

[B46] Thyreau B. Sato K. Fukuda H. Taki Y. (2018). Segmentation of the hippocampus by transferring algorithmic knowledge for large cohort processing. *Med. Image Anal.* 43 214–228. 10.1016/j.media.2017.11.004 29156419

[B47] Tibshirani R. (2011). Regression shrinkage and selection via the lasso: A retrospective. *J. R. Stat. Soc. Ser. B* 73 267–288.

[B48] Trojanowski J. Q. Vandeerstichele H. Korecka M. Clark C. M. Aisen P. S. Petersen R. C. (2010). Update on the biomarker core of the Alzheimer’s Disease Neuroimaging Initiative subjects. *Alzheimers Dement.* 6 230–238. 10.1016/j.jalz.2010.03.008 20451871 PMC2867838

[B49] Wang L. Feng Q. Wang M. Zhu T. Yu E. Niu J. (2021). An effective brain imaging biomarker for AD and aMCI: ALFF in slow-5 frequency band. *Curr. Alzheimer Res.* 18 45–55. 10.2174/1567205018666210324130502 33761855

[B50] Wang Y. Zhao X. Xu S. Yu L. Wang L. Song M. (2015). Using regional homogeneity to reveal altered spontaneous activity in patients with mild cognitive impairment. *Biomed. Res. Int.* 2015:807093. 10.1155/2015/807093 25738156 PMC4337114

[B51] Weiner M. W. Aisen P. S. Jack C. R. Jr. Jagust W. J. Trojanowski J. Q. Shaw L. (2010). The Alzheimer’s disease neuroimaging initiative: Progress report and future plans. *Alzheimers Dement.* 6 202–211.e7. 10.1016/j.jalz.2010.03.007 20451868 PMC2927112

[B52] Whitfield-Gabrieli S. Nieto-Castanon A. (2012). Conn: A functional connectivity toolbox for correlated and anticorrelated brain networks. *Brain Connect.* 2 125–141. 10.1089/brain.2012.0073 22642651

[B53] Wu H. Song Y. Chen S. Ge H. Yan Z. Qi W. (2022). An activation likelihood estimation meta-analysis of specific functional alterations in dorsal attention network in mild cognitive impairment. *Front. Neurosci.* 16:876568. 10.3389/fnins.2022.876568 35557608 PMC9086967

[B54] Xu H. Lv W. Feng H. Du D. Yuan Q. Wang Q. (2020). Subregional radiomics analysis of PET/CT imaging with intratumor partitioning: Application to prognosis for nasopharyngeal carcinoma. *Mol. Imaging Biol.* 22 1414–1426. 10.1007/s11307-019-01439-x 31659574

[B55] Yan C. G. Wang X. D. Zuo X. N. Zang Y. F. (2016). DPABI: Data processing & analysis for (Resting-State) brain imaging. *Neuroinformatics* 14 339–351. 10.1007/s12021-016-9299-4 27075850

[B56] Yang L. Yan Y. Wang Y. Hu X. Lu J. Chan P. (2018). Gradual disturbances of the Amplitude of Low-Frequency Fluctuations (ALFF) and fractional ALFF in alzheimer spectrum. *Front. Neurosci.* 12:975. 10.3389/fnins.2018.00975 30618593 PMC6306691

[B57] Yuan Q. Qi W. Xue C. Ge H. Hu G. Chen S. (2021). Convergent functional changes of default mode network in mild cognitive impairment using activation likelihood estimation. *Front. Aging Neurosci.* 13:708687. 10.3389/fnagi.2021.708687 34675797 PMC8525543

[B58] Zang Y. F. He Y. Zhu C. Z. Cao Q. J. Sui M. Q. Liang M. (2007). Altered baseline brain activity in children with ADHD revealed by resting-state functional MRI. *Brain Dev.* 29 83–91. 10.1016/j.braindev.2006.07.002 16919409

[B59] Zang Y. Jiang T. Lu Y. He Y. Tian L. (2004). Regional homogeneity approach to fmri data analysis. *Neuroimage* 22 394–400. 15110032 10.1016/j.neuroimage.2003.12.030

[B60] Zhang J. Yu C. Jiang G. Liu W. Tong L. (2012). 3D texture analysis on MRI images of Alzheimer’s disease. *Brain Imaging behav.* 6 61–69. 10.1007/s11682-011-9142-3 22101754

[B61] Zhao K. Ding Y. Han Y. Fan Y. Alexander-Bloch A. Han T. (2020). Independent and reproducible hippocampal radiomic biomarkers for multisite Alzheimer’s disease: Diagnosis, longitudinal progress and biological basis. *Sci. Bull.* 65 1103–1113. 10.1016/j.scib.2020.04.003 36659162

[B62] Zheng B. H. Liu L. Z. Zhang Z. Z. Shi J. Y. Dong L. Q. Tian L. Y. (2018). Radiomics score: A potential prognostic imaging feature for postoperative survival of solitary HCC patients. *BMC Cancer* 18:1148. 10.1186/s12885-018-5024-z 30463529 PMC6249916

[B63] Zuo X. N. Di Martino A. Kelly C. Shehzad Z. E. Gee D. G. Klein D. F. (2010). The oscillating brain: Complex and reliable. *Neuroimage* 49 1432–1445. 10.1016/j.neuroimage.2009.09.037 19782143 PMC2856476

[B64] Zwanenburg A. Vallières M. Abdalah M. A. Aerts H. Andrearczyk V. Apte A. (2020). The image biomarker standardization initiative: Standardized quantitative radiomics for high-throughput image-based phenotyping. *Radiology* 295 328–338. 10.1148/radiol.2020191145 32154773 PMC7193906

