# Supplementary material for: Textural features reflecting local activity of the hippocampus improve the diagnosis of Alzheimer’s disease and amnestic mild cognitive impairment: A radiomics study based on functional magnetic resonance imaging
Source: Front Neurosci. 2022 Aug 8;16:970245. doi: 10.3389/fnins.2022.970245 (PMC9393721; doi:10.3389/fnins.2022.970245)
Supplement: Supplementary file 1 [file Data_Sheet_1.docx]

***Supplementary Materials***

**Table S1** | Radiomics features

| **Category** | | **Features** |
| --- | --- | --- |
| **Intensity** | | max, peak, mean, std, var, energy, AUC_CSH, Mean-intensity, Variance-intensity, Skewness-intensity, Kurtosis-intensity, Energy-intensity, Entropy-histogram |
| **Texture** | GLCM | Energy, Entropy, Difference entropy, Sum entropy, Variance1, Variance2, Sum variance, Max Possibility, Contrast, Dissimilarity, Homogeneity1, Homogeneity2, Correlation, DiffVar, Autocorrelation, Cluster prominence, Cluster shade, Cluster tendency, ICM1, ICM2, InVar, IDMN, IDN, Sum Average1, Sum Average2, Agreement |
|  | GLRLM | SRE, LRE, GLN, RLN, RP, LGRE, HGRE, SRLGE, SRHGE, LRLGE, LRHGE, GLV, RLV |
|  | GLSZM | SZE, LZE, GLN, ZSN, ZP, LGZE, HGZE, SZLGE, SZHGE, LZLGE, LZHGE, GLV, ZSV |
|  | NGTDM | Coarseness, Contrast, Busyness, Complexity, Strength |
|  | GLGLM | SGE, LGE, GLF, GaLN, GP, LGGE, HGGE, SGLGE, SGHGE, LGLGE, LGHGE, GrLV, GaLV |
|  | NGLDM | Entropy, Energy, SNE, LNE, NNU |
|  | TS | BWS, MasSpe |
|  | TFC | Coarseness, MeanCovergence, Variance |
|  | TFCCM | Code Entropy, Code Similarity, Contrast, SAM, IDM, Homogeneity, Intensity, Entropy |

Abbreviation: GLCM gray level co-occurrence matrix, GLRLM gray level run length matrix, GLSZM gray level size zone matrix, NGTDM neighborhood gray tone difference matrix, GLGLM gray-level run-length matrix, NGLDM neighboring gray level dependence matrix, TS texture spectrum, TFC texture feature coding, TFCCM texture feature coding method.

**GLCM**: Energy, Entropy, Difference entropy, Sum entropy, Variance1, Variance2, Sum variance, Max Possibility, Contrast, Dissimilarity, Homogeneity1, Homogeneity2, Correlation, DiffVar difference variance, Autocorrelation, Cluster prominence, Cluster shade, Cluster tendency, ICM1 informational measure of correlation1, ICM2 informational measure of correlation2, InVar inverse variance, IDMN inverse difference moment normalized, IDN

inverse difference normalized, Sum Average1, Sum Average2, Agreement

GLRLM: SRE short run emphasis, LRE long run emphasis, GLN gray-level non-uniformity, RLN run-length nonuniformity, RP run percentage, LGRE low gray-level run emphasis, HGRE high gray-level run emphasis, SRLGE short run low gray-level emphasis, SRHGE short run high gray-level emphasis,

LRLGE: long run low gray-level emphasis, LRHGE long run high gray-level emphasis, GLV gray-level variance, RLV run-length variance

GLSZM: SZE small zone emphasis, LZE large zone emphasis, GLN gray-level non-uniformity, ZSN zone-size nonuniformity, ZP zone percentage, LGZE low gray-level zone emphasis, HGZE high gray-level zone emphasis, SZLGE small zone low gray-level emphasis, SZHGE small zone high gray-level emphasis, LZLGE large zone low gray-level emphasis, LZHGE large zone high gray-level emphasis, GLV gray-level variance, ZSV zone-size variance

GLGLM: SGE short gap emphasis, LGE long gaps emphasis, GLF gray level fluctuation, GaLN gap length nonuniformity, GP gap percentage, LGGE Low Gray-Level Gap Emphasis, HGGE High Gray-Level Gap Emphasis, SGLGE Short Gap Low Gray-Level Emphasis, SGHGE Short Gap High Gray-Level Emphasis, LGLGE Long Gap Low Gray-Level Emphasis, LGHGE Long Gap High Gray-Level Emphasis, GrLV Gray-Level Variance, GaLV GapLength Variance.

NGLDM: SNE Small number emphasis, LNE Large number emphasis, NNU number nonuniformity.

TS: BWS black white symmetry, MasSpe Max spectrum.

TFCCM: SAM Second angular moment, IDM inverse difference moment.

**Table S2** | Demographic data and clinical characteristics of the participants from ADNI.

| **Sample size** | **AD (N = 32)** | **MCI (N = 32)** | **NC (N = 35)** | ***Statistic*** | ***P*-value** |
| --- | --- | --- | --- | --- | --- |
| Gender (male: female) | 15:17 | 22:10 | 14:21 | 5.940 | 0.051^a^ |
| Age (years, mean± SD) | 72.444±7.320 | 72.022±8.746 | 74.617±5.850 | 1.215 | 0.301^b^ |
| MMSE | 21.094±2.988 | 27.219±2.352 | 28.943±1.474 | 102.850 | < 0.001^b^ |

^a^*p*-values for sex distribution obtained by the chi-square test; ^b^*p*-value obtained by analysis of variance. Abbreviations: AD, Alzheimer’s disease; aMCI, amnestic mild cognitive impairment; NCs, normal controls.

**Table S3** | The ROC curve of bilateral hippocampal structural images, ALFF in slow-5 frequency band and their combined model.

| **Classifier** | **Model** | **Data set** | **AUC** | **Accuracy** |
| --- | --- | --- | --- | --- |
| AD vs. NCs | T1 | Training | 0.863 | 0.800 |
|  |  | Test | 0.797 | 0.737 |
|  |  | External validation | 0.714 | 0.672 |
|  | ALFF | Training | 0.852 | 0.778 |
|  |  | Test | 0.751 | 0.658 |
|  |  | External validation | 0.667 | 0.672 |
|  | T1+ALFF | Training | 0.867 | 0.844 |
|  |  | Test | 0.797 | 0.658 |
|  |  | External validation | 0.721 | 0.672 |
| aMCI vs. NCs | T1 | Train | 0.741 | 0.727 |
|  |  | Test | 0.687 | 0.714 |
|  |  | External validation | 0.518 | 0.567 |
|  | ALFF | Training | 0.802 | 0.773 |
|  |  | Test | 0.697 | 0.714 |
|  |  | External validation | 0.621 | 0.597 |
|  | T1+ALFF | Training | 0.814 | 0.788 |
|  |  | Test | 0.708 | 0.750 |
|  |  | External validation | 0.639 | 0.687 |

abbreviations: AD, Alzheimer’s disease; aMCI, amnestic mild cognitive impairment; NCs, normal controls; ROC, area under the curve; ALFF, the amplitude of low frequency fluctuation.


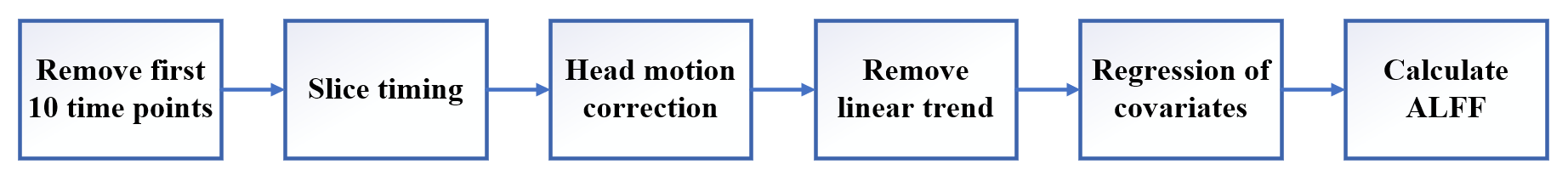


**Figure S1**: The flow chart for calculating ALFF using resting-state fMRI data.

**
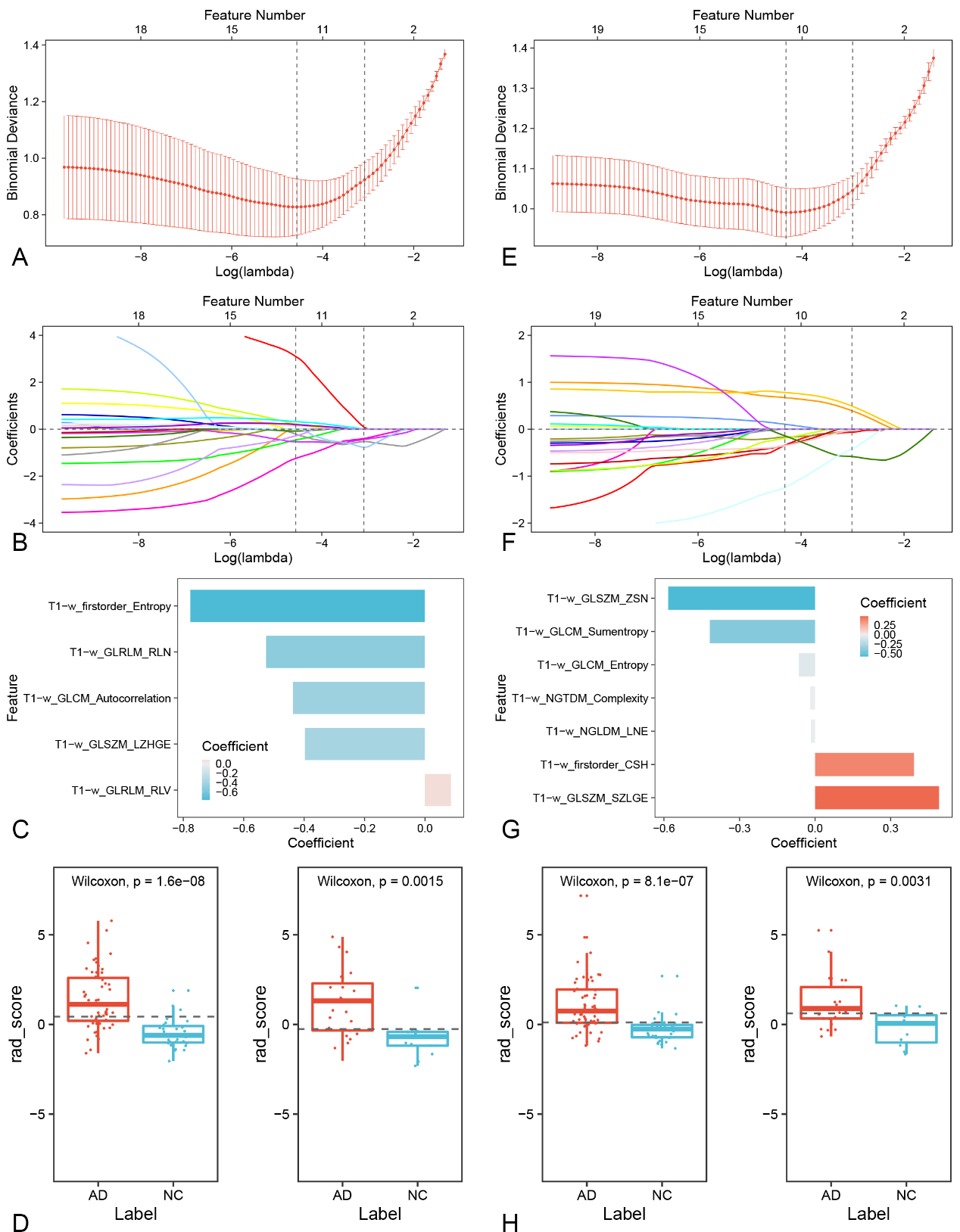
Figure S2**: Feature selection and radiomics signature building based on the hippocampal structural image for AD diagnosis. (A, B, C, D) feature selection and radiomics signature building based on the left hippocampal structural image for AD diagnosis; (E, F, G, H) feature selection and radiomics signature building based on the right hippocampal structural image for AD diagnosis.

**
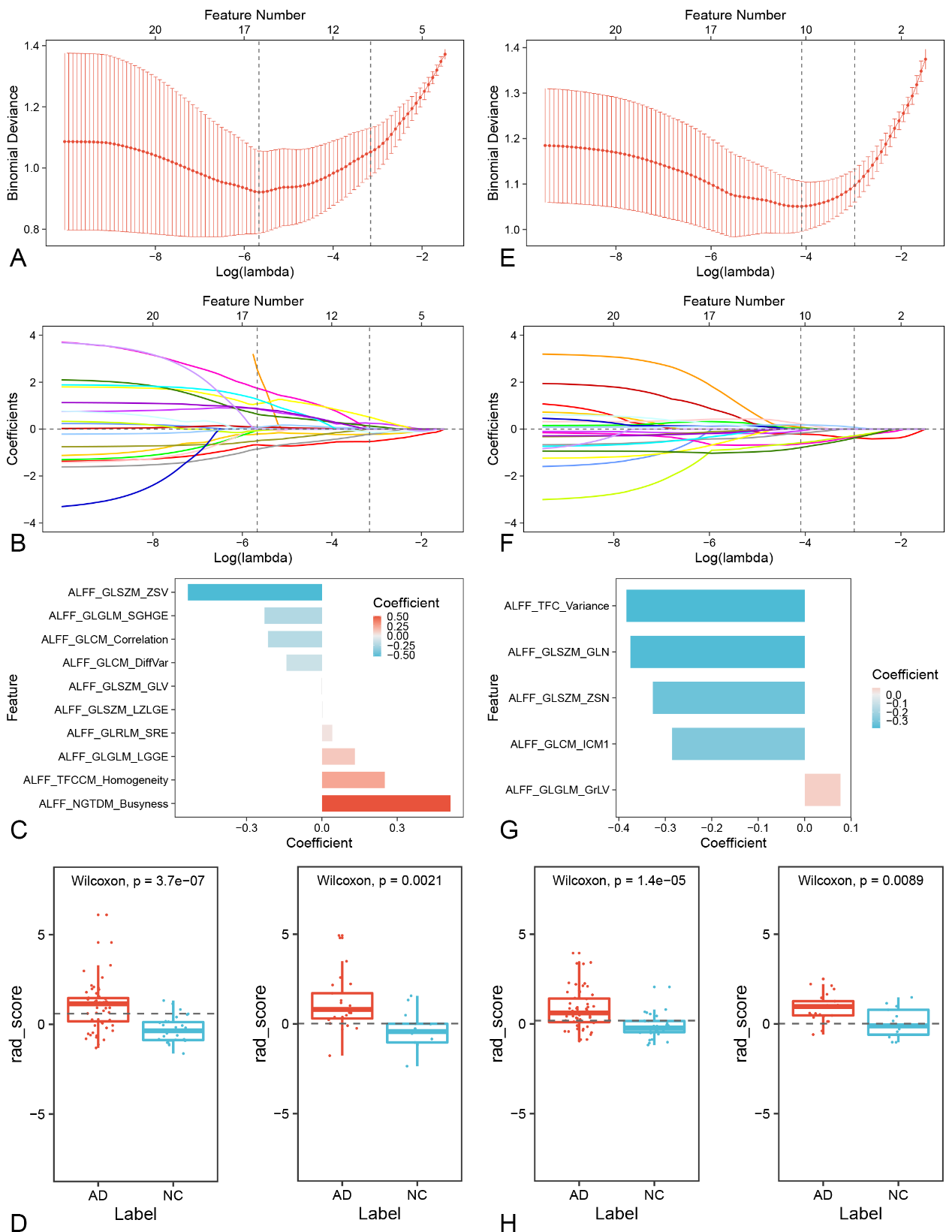
**

**Figure S3**: Feature selection and radiomics signature building based on hippocampal ALFF in the slow-5 frequency band for AD diagnosis. (A, B, C, D) feature selection and radiomics signature building based on left hippocampal ALFF in the slow-5 frequency band for AD diagnosis; (E, F, G, H) feature selection and radiomics signature building based on right hippocampal ALFF in the slow-5 frequency band for AD diagnosis.

**
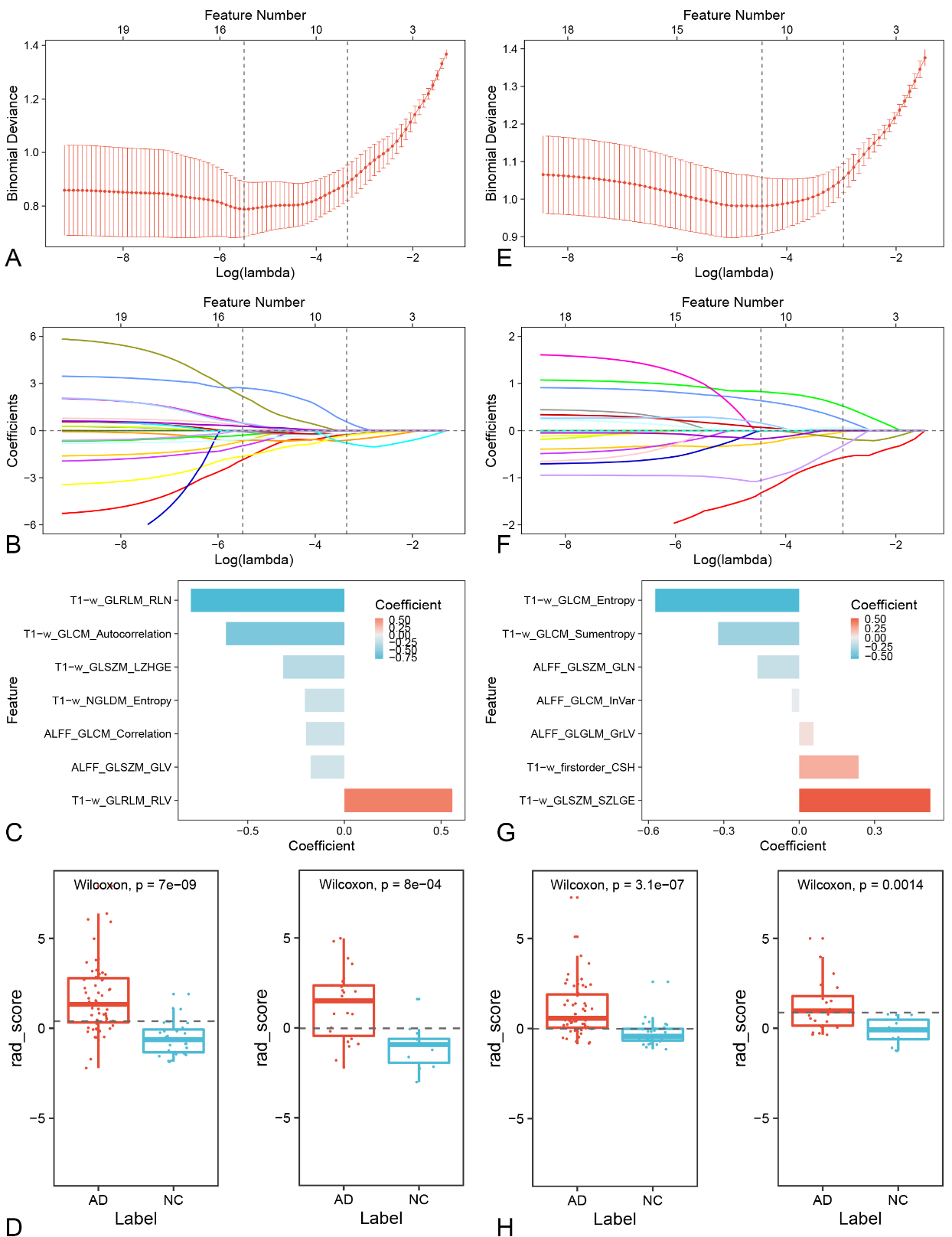
Figure S4**: Feature selection and radiomics signature building based on hippocampal structural image and ALFF in the slow-5 frequency band for AD diagnosis. (A, B, C, D) feature selection and radiomics signature building based on left hippocampal structural image and ALFF in the slow-5 frequency band for AD diagnosis; (E, F, G, H) feature selection and radiomics signature building based on right hippocampal structural image and ALFF for AD diagnosis.


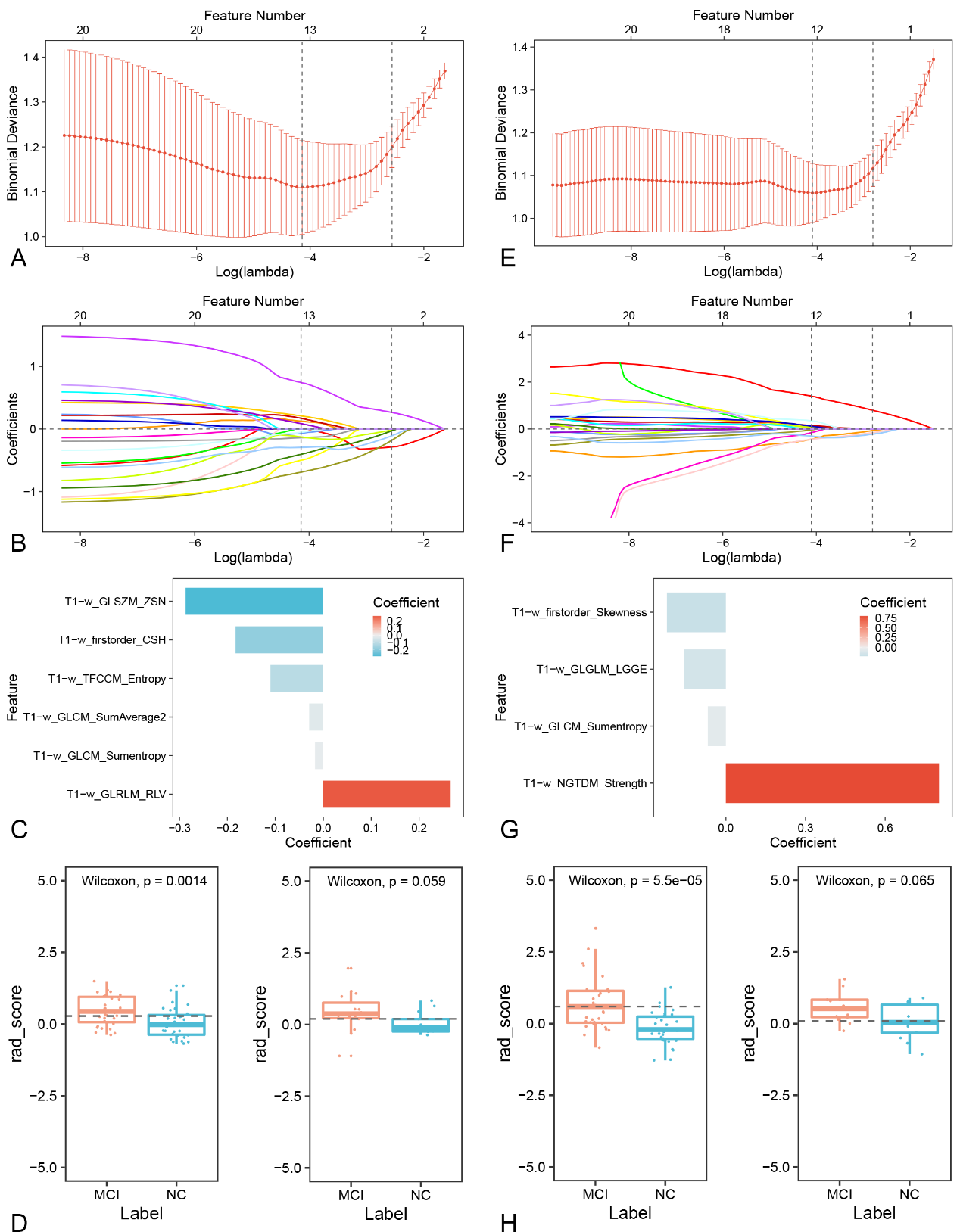


**Figure S5**: Feature selection and radiomics signature building based on the hippocampal structural image for aMCI diagnosis. (A, B, C, D) feature selection and radiomics signature building based on the left hippocampal structural image for aMCI diagnosis; (E, F, G, H) feature selection and radiomics signature building based on the right hippocampal structural image for aMCI diagnosis.


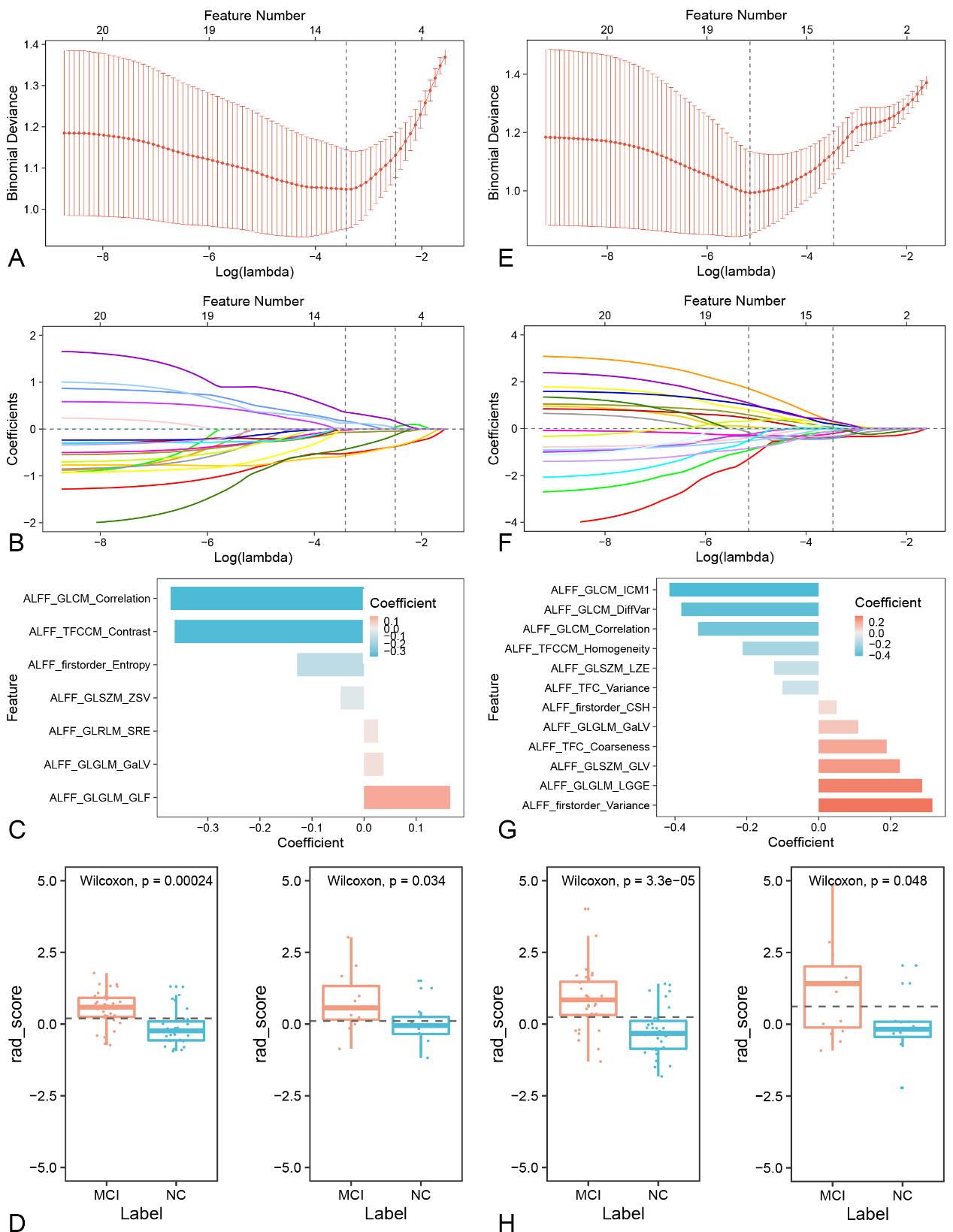


**Figure S6**: Feature selection and radiomics signature building based on hippocampal ALFF in the slow-5 frequency band for aMCI diagnosis. (A, B, C, D) feature selection and radiomics signature building based on left hippocampal ALFF in the slow-5 frequency band for aMCI diagnosis; (E, F, G, H) feature selection and radiomics signature building based on right hippocampal ALFF in the slow-5 frequency band for aMCI diagnosis.

**
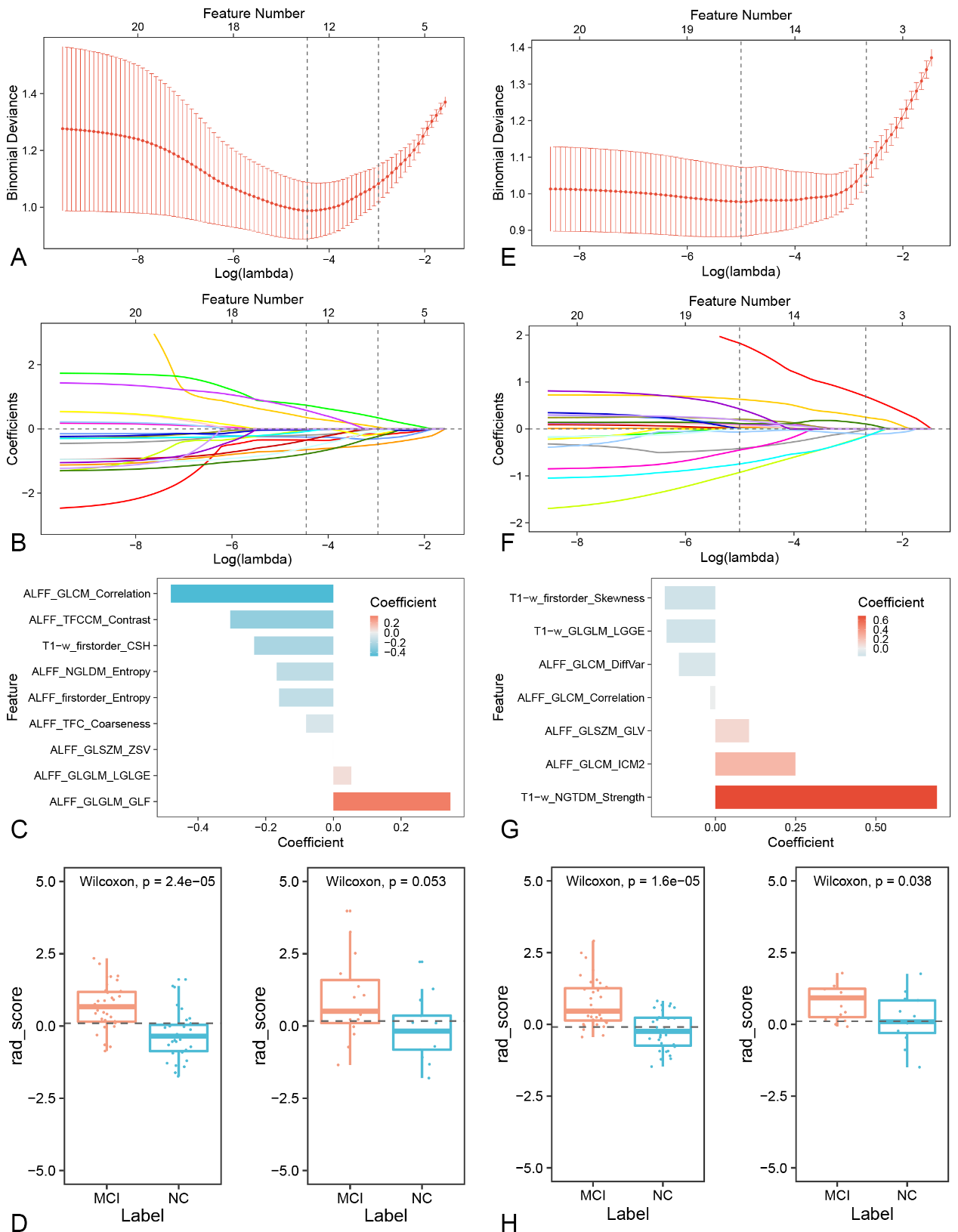
Figure S7**: Feature selection and radiomics signature building based on hippocampal structural image and ALFF in the slow-5 frequency band for aMCI diagnosis. (A, B, C, D) feature selection and radiomics signature building based on left hippocampal structural image and ALFF in the slow-5 frequency band for aMCI diagnosis; (E, F, G, H) feature selection and radiomics signature building based on right hippocampal structural image and ALFF for aMCI diagnosis.
